# Prognostic value of tumor-infiltrating lymphocytes in DCIS: a meta-analysis

**DOI:** 10.1186/s12885-022-09883-9

**Published:** 2022-07-18

**Authors:** Shuang-Ling Wu, Xinmiao Yu, Xiaoyun Mao, Feng Jin

**Affiliations:** Department of Surgical Oncology and Breast Surgery, the First Affiliated Hospital of China Medical University, No. 155, North Nanjing Street, Shenyang, 110001 Liaoning Province China

**Keywords:** Tumor infiltrating lymphocytes (TILs), PD-L1, ductal carcinoma in situ (DCIS), recurrence risk, prognosis

## Abstract

**Background:**

Tumor infiltrating lymphocytes (TILs) have been shown to be associated with the prognosis of breast ductal carcinoma in situ (DCIS). In this systematic review and meta-analysis, we investigated the role of TILs and TIL subsets in predicting the recurrence risk of DCIS.

**Method:**

PubMed, Medline, Web of Science, Embase and Cochrane were searched to identify publications investigating the prognostic role of TILs in DCIS. After study screening, data extraction and risk of bias assessment, a meta-analysis was performed to assess the association between TILs (total TILs, CD4+, CD8+, FOXP3+, PD-L1+ TILs) and the risk of DCIS recurrence.

**Results:**

A pooled analysis indicated that dense stromal TILs in DCIS were associated with a higher recurrence risk (HR 2.11 (95% CI 1.35–3.28)). Subgroup analysis showed that touching TILs (HR 4.73 (95% CI 2.28–9.80)) was more precise than the TIL ratio (HR 1.49 (95% CI 1.11–1.99)) in estimating DCIS recurrence risk. Moreover, the prognostic value of TILs seemed more suitable for patients who are diagnosed with DCIS and then undergo surgery (HR 2.77, (95% CI 1.26–6.07)) or surgery accompanied by radiotherapy (HR 2.26, (95% CI 1.29–3.95)), than for patients who receive comprehensive adjuvant therapies (HR 1.16, (95% CI 1.35–3.28)). Among subsets of TILs, dense stromal PD-L1+ TILs were valuable in predicting higher recurrence risk of DCIS.

**Conclusion:**

This systematic review and meta-analysis suggested a non-favorable prognosis of TILs and stromal PD-L1+ TILs in DCIS and indicated an appropriate assessment method for TILs and an eligible population.

## Introduction

Widespread use of mammographic screening has largely increased the detection rate of breast ductal carcinoma in situ (DCIS), which accounts for 20–25% of newly diagnosed breast cancer [1]. Theoretically, surgical dissection is adequate for DCIS treatment. For patients receiving surgery alone, the local recurrence risk ranges from 10.5 to 18% [2], and invasive cancer events occur in 19.2% of high-grade DCIS patients [3]. In addition, the mortality risk for patients who experience recurrence is 18 times higher than that for those who do not [4]. Thus, it is crucial to identify risk factors in predicting the recurrence risk of DCIS in order to carry out appropriate management strategies.

DCIS is a heterogeneous disease, and its recurrence is a complex process caused by the coevolution of cancer cells and the immune microenvironment. Cells of the tumor microenvironment mainly include tumor infiltrating lymphocytes (TILs), NK cells, macrophages, dendritic cells and myeloid lineage cells [5]. In recent years, accumulating evidence has suggested that TILs in the immune microenvironment are associated with better prognosis in basal-like and HER2-positive invasive breast cancers [6]. In contrast to invasive breast cancer, the role of total TILs and specific subtypes of TILs in DCIS remain ambiguous.

Tumor-infiltrating lymphocytes are an important component of tumor environment and play an essential role in cancer progression. In DCIS, dense TILs were shown to be associated with many clinical factors, including younger age, higher tumor grade, comedo necrosis and molecular subtype [7]. However, it remains ambiguous whether dense TILs in DCIS are associated with aggressive tumor features and tumor recurrence risk. A few previous studies have reported that there was no significant difference between dense and sparse TILs groups in tumor recurrence [8, 9]. Some other studies recently put forward that dense TILs are associated with higher recurrence risk [10–13]. Moreover, some research proposed that the value of TILs in predicting recurrence risk is associated with TIL assessment methods [14] and included patient therapy strategies [15].

Along with total TILs, different subsets of TILs also exhibit diverse functions in cancer progression. The TILs in DCIS are generally composed of CD3+ T cells, followed by CD4+ T cells, CD8+ T cells, CD20+ B cells and FOXP3+ regulatory T cells (Tregs) [16]. Among these cells, it is now believed that CD8+ and CD4+ T cells are involved in the effective immune response, and FOXP3+ regulatory T cells (Tregs) are associated with the suppression of antitumor immunity [17]. However, the exact prognostic role of each subset of lymphocytes in DCIS has not yet been clarified. In addition to the above subsets, the expression of PD-L1 in immune cells is also associated with DCIS subtypes and their recurrence [18]. Considering that the PD-1/PD-L1 axis is effective in triple-negative invasive breast cancer immunotherapy [19], the prognostic and therapeutic value of PD-L1 in DCIS remains to be further investigated.

In order to illustrate whether TILs have prognostic value in DCIS, we performed a systematic review and meta-analysis to investigate the prognostic roles of TILs and CD4+, CD8+, FOXP3+ and PD-L1 TIL subtypes in DCIS. We aimed to identify potential pathological biomarkers about TILs and TIL subsets in DCIS that can be used to predict patient recurrence risk.

## Method

The present systematic review and meta-analysis were performed in accordance with the Preferred Reporting Item for Systematic Reviews and Meta-Analyses (PRISMA) statement.

### Search strategy and study selection

We performed an extensive literature search of electronic databases including Pubmed, Medline, Web of Science, Excerpta Medica Database (Embase) and Cochrane up to 5 April 2021 by two investigators. The search strategy was in line with published articles, and the following determinant domains were used: (“Ductal Carcinoma in Situ” OR “DCIS” OR “Intraductal Carcinoma”) AND (“Tumor infiltrating lymphocyte” OR “Infiltrating lymphocyte” OR “Immune” OR “Immune cell” OR “Immunology” OR “TILs” OR “TIL assessment” OR “lymphocyte” OR “CD4” OR “CD8” OR “FOXP3” OR “PD-L1”) AND (“Prognosis” OR “Survival” OR “recurrence”). In addition, All the proceedings in scientific meetings and references of the selected articles were searched to identify associated data. The title and abstract of each study in the search were scanned by two independent reviewers, clearly irrelevant studies were excluded.

### Inclusion and exclusion criteria

Inclusion criteria were as follows: (1). Patients diagnosed with DCIS or DCIS with micro-invasive lesions confirmed with pathological examination; (2). Total TILs and specific subtypes of TILs were measured according to HE and IHC staining; (3). Original research articles; (4). Correlation of TILs with tumor recurrence was illustrate with Hazard Ratio (HR) and a 95% confidence interval (95% CIs).

Exclusion criteria were as follows: (1). Overlapping articles or repeat analysis; (2). Studies lacking sufficient data for assessing Hazard Ratio (HR) and a 95% confidence interval (95% CIs); (3). Study with missing data and unavailable HR; (4). Types of Case reports, reviews, letters, comments and nonclinical studies.

### Data extraction

All the data from candidate studies were evaluated and extracted by two independent investigators. Disagreements in data extraction were discussed and resolved by consensus. The following data were obtained from each study: year of publication, first author, country of the population studied, pathology of studied samples, total number of included cases, method of TIL’s detection, cutoff of dense TIL’s classification, cell type of studied TILs, treatment strategy, time of follow-up, deadline (type of recurrence).

No restrictions regarding study design, observational studies, including cohort study and case-control study, were included. The patients diagnosed with DCIS, DCIS mixed with micro-invasive breast cancer or DCIS mixed with invasive breast cancer were all included without restriction of patients’ clinical characteristics and patients’ adjuvant treatments. The total TILs were assessed with HE staining, and TIL subsets as well as PD-L1+ tumor cells were assessed with immunohistochemical staining. The level of the total TILs, CD4+ TILs, CD8+ TILs, FOXP3+ TILs, stromal PD-L1+ TILs and PD-L1+ tumor cells were evaluated with TILs percentage or the number of touching-TILs. Recurrence was defined as any in situ or invasive carcinoma relapse in ipsilateral breast, contralateral breast, axilla, or chest and distant metastasis.

### Quality assessment

The Newcastle-Ottawa Scale (NOS) was used to assess the quality of each included study and the risk of bias in each study. The quality assessment was performed by two investigators independently. The NOS consists of three items including selection (0–4 points), comparability (0–2 points), and outcome assessment (0–3 points). NOS scored more than 7 were assigned as high-quality studies.

### Statical analysis

The meta-analysis calculated the pooled HR and corresponding 95% CIs to evaluate the prognostic value of TILs in DCIS. All statistical analyses were performed with STATA version 15. Higgins I-squared statistic were used to estimate the heterogeneity of the included studies. Random-effect model was adopted in our analysis and heterogeneity analysis was assessed by *I*^2^ and P heterogeneity (*P* < 0.10 or *I*^2^ > 50% was indicative of statistically significant heterogeneity). Sensitivity analysis and meta-regression were used to explore the origin of heterogeneity. Publication bias was assessed by Egger test and Begg funnel plot. All statistical tests were two-sided, *P* value < 0.05 was considered statistically significant.

## Results

### Literature research

**Fig. 1:**
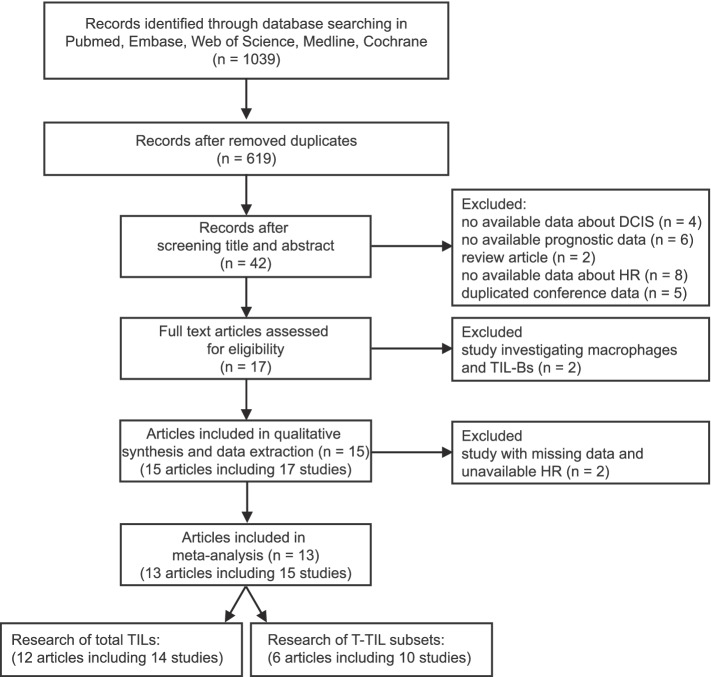
Flow diagram of study selection and identification

A total of 1039 records were searched in Medline, PubMed, Embase, Cochrane and Web of Science. After excluding duplicates, 619 records remained. Next, we screened the titles and abstracts of the 619 papers, and only 42 papers were included for further full-text review. Among these papers, 20 papers were excluded because they did not provide relevant data in estimating TILs in DCIS, and another 5 were conference abstracts that displayed the same data as other included papers. Next, 2 papers were excluded because they focused on infiltrating macrophage cells and TIL-Bs in DCIS； and 2 articles were excluded because their missing data and unavailable HR. Finally,13 articles including 15 sets of studies were used for following meta-analysis. Among these 13 studies, 12 studies including 14 studies investigated the prognostic role of total TILs in DCIS, and 6 articles containing 10 studies explored the value of TIL subsets in DCIS (Fig. 1).

### Included studies’ characteristics

Detailed characteristics of the included articles are listed in Table 1. These articles were conducted in the United States (2), China (1), Europe (1), Australia (2), the United Kingdom (3), Singapore (1), the Netherlands (1), Italy (1) and Belgium (1), including approximately 4843 participations. All 13 articles were retrospective cohort studies, 1 of the 13 was a conference abstract, and the others were full-reported articles. Among these, 12 articles, which included 14 sets of studies, evaluated the relationship between TILs and DCIS recurrence, and 6 articles investigated the prognostic value of TIL subtypes (CD4+, CD8+, FOXP3+, PD-L1) in DCIS.

**Table 1:** Characteristics of included studies in meta-analysis

Year	First author	Country	Type of DCIS	Number of participants	TIL’s detection method	Cutoff of TIL’s point	Subtype of TILs	Treatment	Median follow up	Outcome	Reference
2006	Gaynor J. Bates et al.	UK	p-DCIS	62	IHC	15	FOXP3+ TILs	S & AT (RT/ HT)	5.4 y	AR	[20]
2016	G. Pruneri et al.	Europe	DCIS	1488 (set1: *n* = 1391; set2: *n* = 620)	HE	set1: < 1% vs. 35–50%	total p-TILs	S & AT (RT/ HT)	8.2 y	IR	[8]
						set2: < 1% vs. > 50%					
2015	Elizabeth Thompson et al.	USA	p-DCIS & i-DCIS	total p-TILs (n = 27) PD-L1+ TILs (*n* = 27)	HE & IHC	50%	total p-TILs	S & AT	80 m	IR	[9]
							PD-L+ TILs				
2017	Shona Hendry et al.	Australia	p-DCIS	total p-TILs (*n* = 138) PD-L1+ TILs (*n* = 79) PD-L1+ TC(n = 79)	HE & IHC	TILs: 2%. PD-L1: 1%	total p-TILs	S & AT (RT/ HT)	TILs: 89 m PD-L1:39 m	IR	[7]
							PD-L1+ TILs				
							PD-L1+ TC				
2018	Michael S. Toss et al.	UK	training set:	training set	HE	training set: 5%	training set:	training set:	training set: 161 m	IR	[14]
			p-DCIS	(*n* = 150)			total p-TILs	S/ S & RT			
			validation set:	validation set		validation set: 20	validation set:	validation set:	validation set: 109 m		
			p-DCIS	(*n* = 534)			total t-TILs	S/ S & RT			
2019	Rajiv Dave et al.	Australia	p-DCIS	423	HE	5%	total p-TILs	BCS	119 m	IR	[21]
2020	Marie Colombe Agehozo et al.	Netherlands	p-DCIS	total p-TILs (*n* = 466) PD-L1+ TILs (*n* = 115) PD-L1+ TC(n = 115)	HE & IHC	30%	total p-TILs	BCS/ MAS	98 m	IR	[22]
							PD-L1+ TILs				
							PD-L1+ TC				
2019	Farbod Darvishian et al.	USA	p-DCIS	69	HE	45%	total p-TILs	BCS	6.7 y	IR	[11]
2019	Mieke Van Bockstal et al.	Belgium	p-DCIS	211	HE	50%	total p-TILs	S & AT (RT/ HT)	124 m	IR	[10]
2020	Aye Aye THIKE et al.	Singapore	DCIS	total p-TILs (*n* = 198) CD4+ TILs (n = 198)	HE & IHC	20%	total p-TILs	S (BCS/ MAS)	TILs: 7.9 y CD4+: 7.2 y	AR	[23]
							CD4+ TILs				
2020	Michael S. Toss et al.	UK	p-DCIS	total p-TILs (*n* = 508) FOXP3+ TILs(*n* = 406) PD-L1+ TILs (*n* = 383) CD4+ TILs (*n* = 403) CD8+ TILs (n = 402)	HE & IHC	20	total t-TILs	BCS	unknown	IR	[12]
							FOXP3+ TILs				
							PD-L1+ TILs				
							CD4+ TILs				
							CD8+ TILs				
2020	Alberto Farolfi et al.	Italy	p-DCIS	496	HE	5%	total p-TILs	S/ S & RT	56.4 y	IBE	[15]
2021	Fei-Fei Xu et al.	China	p-DCIS &	135	HE	5	total t-TILs	BCS/ BCS & RT	53 m	IR	[13]
			m-DCIS								

*UK* United Kingdom, *USA* United States of America, *p-DCIS* pure DCIS, *i-DCIS* pure DCIS mixed with IDC, *m-DCIS* pure DCIS mixed with microinvasive cancer, *HE* Hematoxylin and eosin staining, IHC Immunohistochemistry staining, *TILs* Tumor-infiltrating lymphocytes, *vt-TILs* Stromal touching TILs, *p-TILs* percentage of stromal TILs, *TC* Tumor cell, *S* Surgery, *BCS* Breast conserving surgery, *MAS* Mastectomy, *AT* Adjuvant therapy, *RT* Radiation therapy, *HT* Hormonal therapy, *IR* Lipsilateral recurrence; *AR* All kinds of recurrence, *IBE* Ipsilateral breast cancer events

### Study quality and risk of bias

**Fig. 2:**
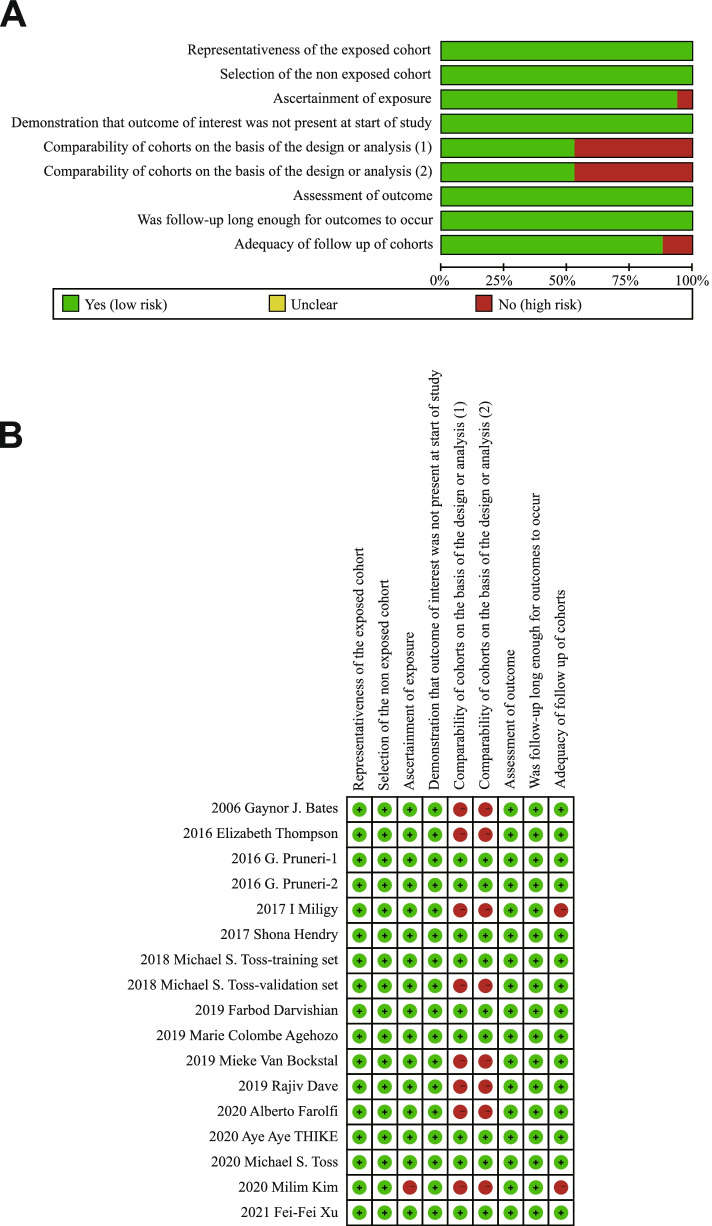
Risk of bias assessment of included studies. **A** Risk of bias graph: review authors’ judgments about each risk of bias item presented as percentages across all included studies; **B** Risk of bias summary: review authors’ judgments about each risk of bias item for each included study

After full-text review, we performed critical assessment for each study by NOS, and the quality of each study is summarized in Fig. 2. Most of the studies exhibit excellent quality with more than six stars. Two studies scored fewer than 7 stars due to missing data, unclear TIL assessment method and univariate analysis. Studies with missing data and unavailable HR were excluded from our meta-analysis; therefore, 15 studies were eligible for inclusion in the meta-analysis.

### The value of total TILs in the recurrence of DCIS

**Fig. 3:**
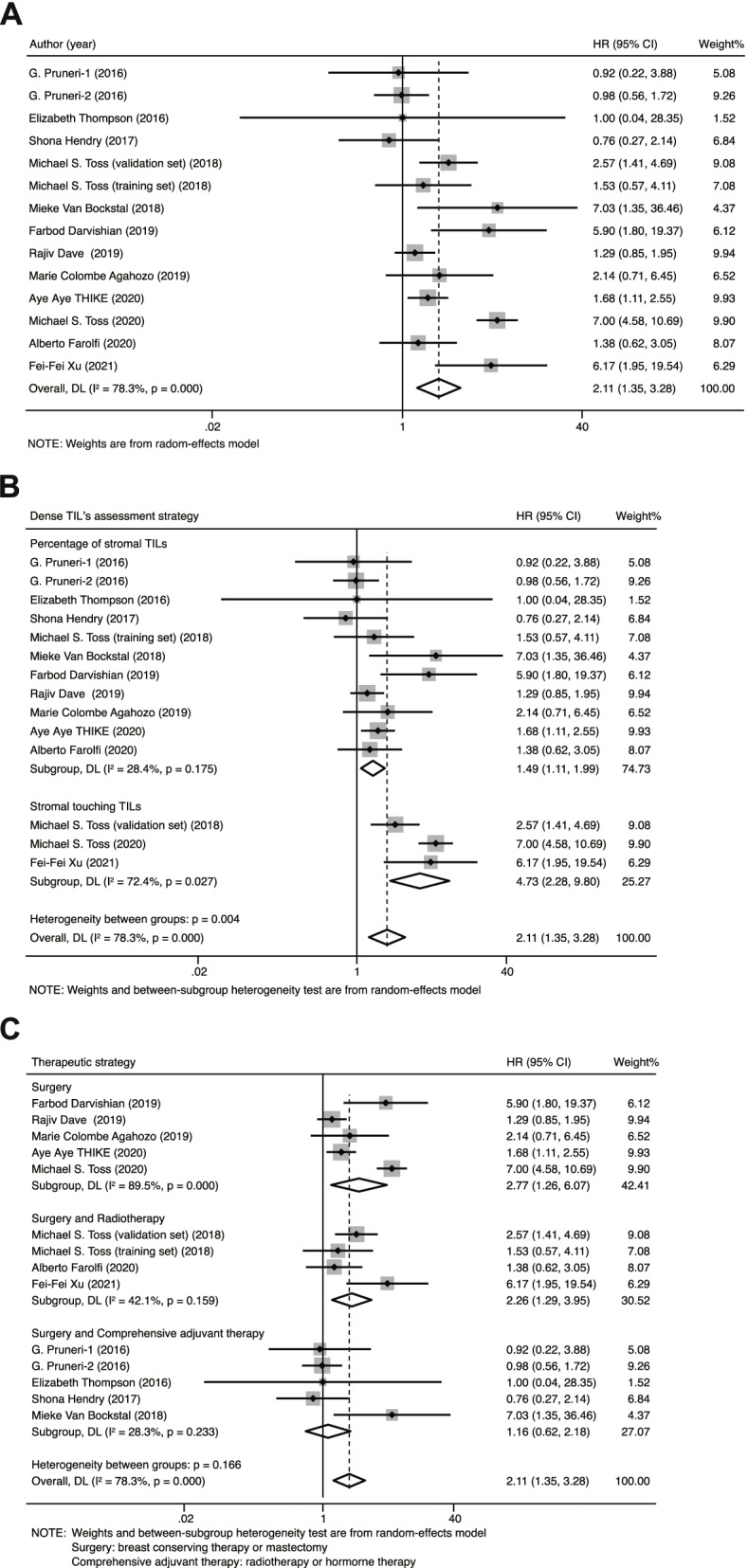
Forest plots of the prognostic value of TILs in patients diagnosed with DCIS. **A** Forest plots of prognostic value of total TILs in DCIS; **B** Forest plots of prognostic value of TILs assessed with different strategies on recurrence risk of DCIS; **C** Forest plots of prognostic value of TILs on patients who receiving different therapies

A total of 14 studies in 12 sets of articles containing 4843 patients were included in our meta-analysis to evaluate the prognostic value of total TILs in DCIS. The results showed that dense TILs in DCIS indicates a higher recurrence risk. The pooled HR was 2.11 (95% CI, 1.35–3.28) for the total TIL level (dense vs. sparse), with statistically significant heterogeneity (*I*^2^ = 78.3%, *P* = 0.000) (Fig. 3A).

To further investigate the heterogeneity in our meta-analysis, we performed meta-regression and subgroup analyses (Table 2). Through meta-regression analysis, we identified “TIL assessment method” as the main cause of heterogeneity (P > |t| = 0.005). Furthermore, in subgroup analysis, we also observed that the assessment method of TILs in different studies may affect the prognostic value of TILs in DCIS. The pooled HR for 11 studies (*N* = 3666) using TIL ratio classification was 1.49 (1.11–1.99), with no obvious heterogeneity between the results of the studies (*I*^2^ = 28.4%, *P* = 0.175). In the other 3 studies (*N* = 1177) that employed touching-TIL classification, the pooled HR was 4.73 (2.28–9.8), with substantial heterogeneity (*I*^2^ = 72.4%, *P* = 0.027). The pooled HR indicated that stromal touching TILs in DCIS were associated with recurrence more closely than the stromal TIL ratio (Fig. 3B). In addition, we observed that the therapeutic strategy is also significant for the evaluation of the prognostic value of TILs. The pooled HRs for those patients who underwent surgery only or surgery accompanied by radiotherapy were 2.77 (95% CI: 1.26–6.07, *I*^2^ = 89.5%, *P* = 0.00) and 2.26 (95% CI: 1.29–3.95, *I*^2^ = 42.1%, *P* = 0.159), which analyzed with 5 (*N* = 1690) and 4 (*N* = 1315) studies, respectively. Five studies (*N* = 1864) were used to investigate the prognostic role of TILs in those patients who experienced comprehensive adjuvant therapy, no prognostic effect on recurrence risk was observed with an HR of 1.16 (95% CI: 0.62–2.18, *I*^2^ = 28.3%, *P* = 0.233) (Fig. 3C). The value of TILs in predicting DCIS recurrence is more suitable for patients who receive surgery only or surgery accompanied by radiotherapy.

**Table 2:** Summary of the prognostic value of TILs obtained from the subgroup analysis including dense TIL’s assessment, Deadline, Variable, Location of TILs, Cutoff of TILs, Therapeutic approach, Pathology and subsets of TILs

Subgroup	Number of studies	Number of participants	Random-effects model		Fixed-effects model		Heterogeneity	
			HR (95%CI)	***P***	HR (95%CI)	***P***	***I***2	***Ph***
**Dense TIL’s assessment**	14	4843	2.11 (1.35–3.28)	0.001	2.13 (1.77–2.55)	0.001	78.30%	0
percentage of stromal TILs	11	3666	1.49 (1.11–1.99)	0.008	1.45 (1.16–1.80)	0	28.40%	0.175
number of touching TILs	3	1177	4.73 (2.28–9.80)	0	5.1 (3.66–7.11)	0	72.40%	0.027
**Outcome**	14	4843	2.11 (1.35–3.28)	0.001	2.13 (1.77–2.55)	0	78.30%	0
ipsilateral recurrence	12	4149	2.26 (1.31–3.91)	0.004	2.33 (1.89–2.88)	0	80.60%	0
all kinds of recurrence	2	694	1.61 (1.11–2.33)	0.011	1.61 (1.11–2.33)	0.011	0	0.662
**Variable**	14	4335	2.11 (1.35–3.28)	0.001	2.13 (1.77–2.55)	0	78.30%	0
univariate analysis	5	1307	1.42 (1.02–1.99)	0.039	1.42 (1.02–1.99)	0.039	0.00%	0.42
multivariate analysis	9	3028	2.30 (1.27–4.17)	0.006	2.52 (2.03–3.14)	0	83.40%	0
**Location of TILs**	14	4843	2.11 (1.35–3.28)	0.001	2.13 (1.77–2.55)	0	78.30%	0
touching TILs	2	204	6.04 (2.64–13.81)	0	6.04 (2.64–13.81)	0	0	0.958
stromal TILs	12	4639	1.81 (1.13–2.90)	0.013	2.02 (1.67–2.43)	0	79.40%	0
**Cutoff of TILs**	14	5366	2.11 (1.35–3.28)	0.001	2.13 (1.77–2.55)	0	78.30%	0
1–5%	6	2625	1.24 (0.90–1.70)	0.186	1.24 (0.90–1.70)	0.186	0	0.933
6–30%	2	664	1.73 (1.17–2.56)	0.006	1.73 (1.17–2.56)	0.006	0	0.69
31–50%	3	900	3.03 (0.71–12.97)	0.134	1.57 (0.97–2.56)	0.068	81.20%	0.005
counts (5 or 15 or 20)	3	1177	4.73 (2.28–9.80)	0	5.10 (3.66–7.11)	0	72.40%	0.027
**Therapeutic approach**	14	4869	2.11 (1.35–3.28)	0.001	2.13 (1.77–2.55)	0	78.30%	0
breast conserving therapy or mastectomy	5	1690	2.77 (1.26–6.07)	0.011	2.51 (1.99–3.17)	0	89.50%	0
breast conserving therapy + radiotherapy	4	1315	2.26 (1.29–3.95)	0.004	2.24 (1.49–3.35)	0	42.10%	0.159
surgery + adjuvant therapies (hormonal therapy, radiotherapy)	5	1864	1.16 (0.62–2.18)	0.645	1.07 (0.69–1.68)	0.753	28.30%	0.233
**Pathology**	14	4843	2.11 (1.35–3.28)	0.001	2.13 (1.77–2.55)	0	78.30%	0
pure DCIS	9	2995	2.37 (1.31–4.28)	0.004	2.54 (2.02–3.18)	0	81.90%	0
pure DCIS & pure DCIS mixed with microinvasive or invasive breast cancer	2	162	5.03 (1.63–15.52)	0.005	5.09 (1.71–15.13)	0.003	1.60%	0.313
DCIS	3	1686	1.32 (0.88–1.97)	0.184	1.36 (0.98–1.89)	0.064	22.60%	0.275
**subset of TILs**	10	2190	2.29 (1.31–3.99)	0.003	2.55 (2.06–3.16)	0	77.80%	0
CD4+ TILs	2	601	1.98 (1.14–3.44)	0.015	1.97 (1.31–2.96)	0.001	45.80%	0.174
CD8+ TILs	1	402	0.90 (0.47–1.71)	0.747	0.90 (0.47–1.71)	0.747	0.00%	–
FOXP3+ TILs	2	468	1.83 (1.23–2.70)	0.003	1.83 (1.23–2.70)	0.003	0.00%	0.382
PD-L1+ TILs	5	719	6.21 (4.26–9.06)	0	6.21 (4.26–9.06)	0	0.00%	0.708

### Different subtypes of TILs play different roles in the recurrence of DCIS

**Fig. 4:**
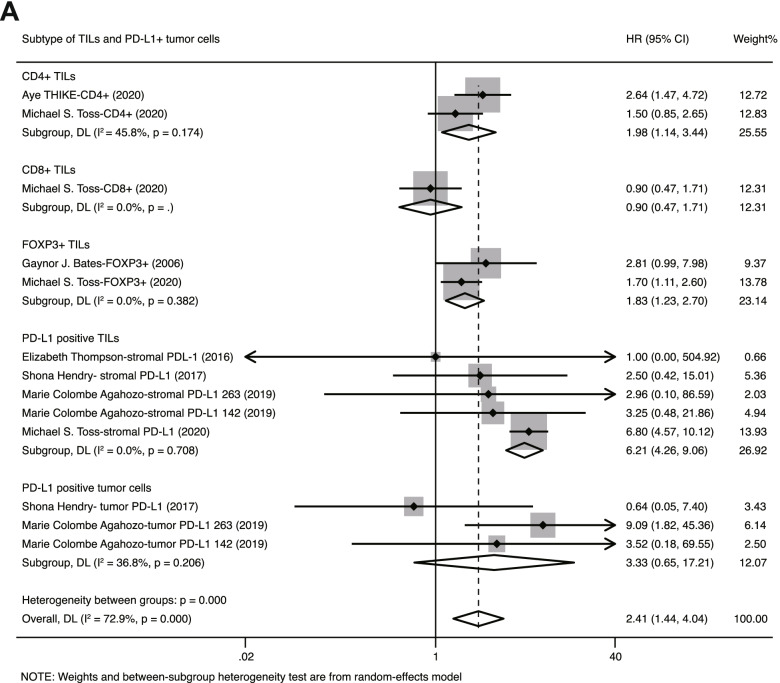
Forest plots of the prognostic value of different subsets of T-TILs and PD-L1+ tumor cells in DICS

Aside from total TILs, we also investigated the prognostic role of CD4+, CD8+, FOXP3+ and PD-L1+ TILs in DCIS. There were 2 studies (*N* = 601) investigating CD4+ TILs, and 2 studies (*N* = 468) investigating FOXP3+ TILs. The pooled HRs of CD4+ and FOXP3+ TILs were estimated to be 1.98 (95% CI: 1.44–3.44) and 1.83 (95% CI: 1.23–2.70), respectively, with no considerable heterogeneity between studies (CD4+: *I*^2^ = 45.8%, *P* = 0.174; FOXP3+: *I*^2^ = 0%, *P* = 0.382). This indicates that dense CD4+, FOXP3+ TILs in DCIS are associated with a higher recurrence risk. In addition to CD4+ and FOXP3+ TILs, we also evaluated the prognostic value of PD-L1+ TILs in DCIS with 5 studies (*N* = 719). The pooled HR for stromal PD-L1 TILs was 6.21 (95% CI: 4.26–9.06, *I*^2^ = 0.0%, *P* = 0.708). Considering that some studies observed positive expression of PD-L1 in intraductal cancer cells in DICS, we further investigated the association between PD-L1+ tumor cells and the recurrence risk of DCIS with 3 studies (*N* = 309). The pooled HR for PD-L1+ tumor cells was 3.33 (95% CI: 0.65–17.21), without apparent heterogeneity (*I*^2^ = 36.8%, *P* = 0.206). Through the above integrated analysis, we observed that both PD-L1+ TILs and PD-L1+ tumor cells are associated with the recurrence risk of DCIS. Regarding CD8+ TILs, an insufficient number of studies provided data to perform a meta-analysis, and only 1 study (*N* = 402) with such data provided showed no significant association between CD8+ TILs and patient recurrence (HR 0.90, 95% CI: 0.47–1.71) (Fig. 4). Taken together, CD4+, FOXP3+, PD-L1 TILs and PD-L1 tumor cells possess the potential to predict the recurrence risk of DCIS, and the stromal PD-L1 is more valuable than the others in evaluating DCIS recurrence risk.

### Sensitivity analysis

**Fig. 5:**
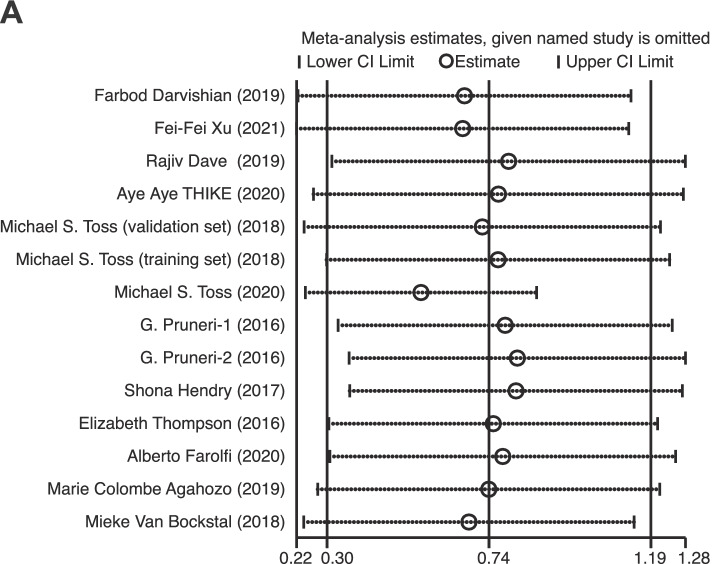
Sensitivity analysis of the meta-analysis of total TILs in DCIS

Sensitivity analysis was performed in our study to estimate the effect of each study on pooled HR by consecutive deletion of each study. The results show that no eligible study exhibited a significant influence on the pooled HR of total TILs (Fig. 5).

### Publication bias

**Fig. 6:**
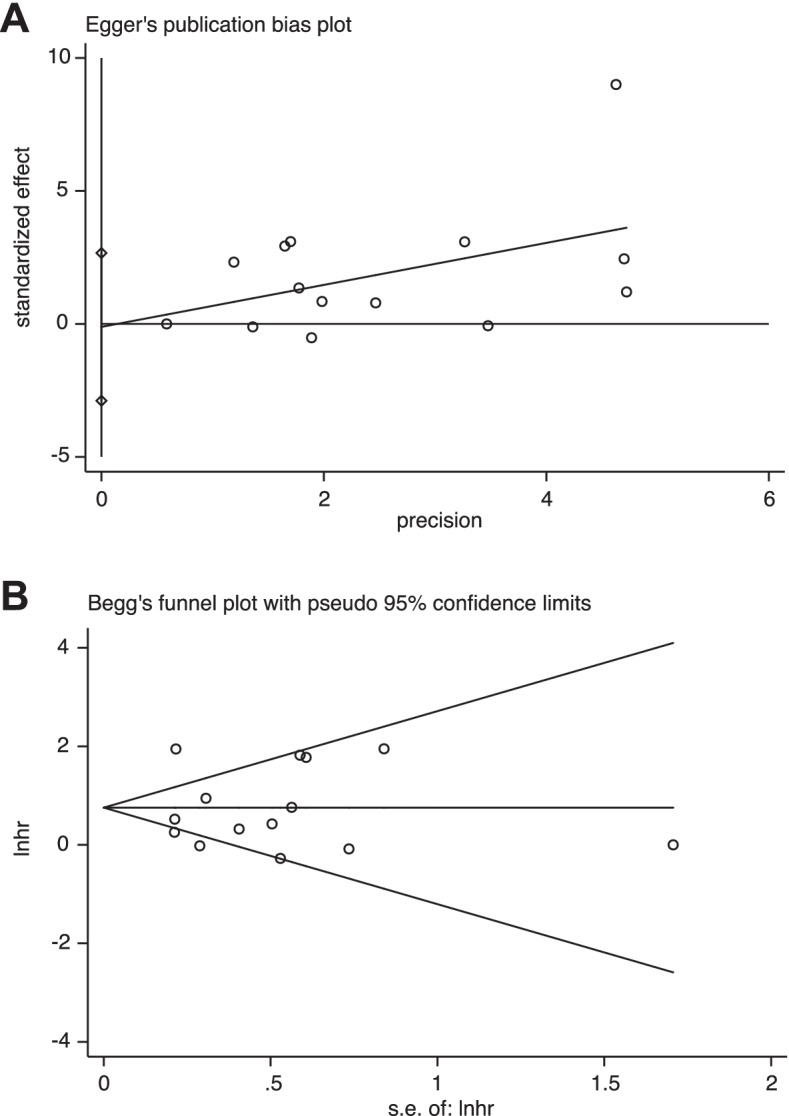
Funnel plots of potential publication bias with Egger’s test (**A**) and Begg’s test (**B**)

Funnel plots display symmetrical distribution and did not indicate any obvious publication bias affecting the HR for cancer recurrence in the included studies (P_Begg_ = 0.228, P_Egger_ = 0.931) (Fig. 6A-B).

## Discussion

Some studies have investigated the prognostic role of tumor infiltrating lymphocytes (TILs) in ductal carcinoma in situ (DCIS), however it is still controversial whether total TILs and subtypes of TILs can indicate recurrence risk of DCIS. Therefore, we performed a systematic review and meta-analysis to evaluate the prognostic value of total TILs and some subsets of TILs, including CD4+, CD8+, FOXP3+ and PD-L1, in DCIS.

Our meta-analysis performed the integrative analysis of TILs with 14 reports, with 4843 patients, which revealed that dense stromal TILs in DCIS indicates higher recurrence risk (HR: 2.11, 95% CI: 1.35–3.28, *p* < 0.001, *I*^2^ = 78.3%). Subgroup analyses showed that touching TILs are more associated with patient recurrence-free survival compared with the stromal TIL ratio. Moreover, the prognostic value of TILs is influenced by the treatment strategies adopted by patients. Only in the subgroup of patients who underwent surgery or surgery accompanied by radiotherapy, TILs are valuable in evaluating patients’ recurrence risk. Subsequent analysis of TIL subsets suggested that PD-L1-positive stomal lymphocytes in DCIS are correlated with recurrence risk more closely than the other subset of TILs.

Through comparative analysis, our observation about the enrichment of TILs in DCIS is generally consistent with invasive breast cancer. Compared with luminal breast cancer, high TILs are more frequent in TNBC and HER-2, which are biologically more aggressive, with high immunogenic characteristics. TILs are considered to reflect the immunogenicity of tumor, and previous study proposed that aggressive tumors may be more immunogenic [24]. Therefore, it was speculated that TILs may be more likely to react to aggressive clones. Additionally, it was observed that the unfavorable prognostic role of TILs in DCIS is in line with luminal breast cancer rather than TNBC [25]. Considering that TILs are the most important immune cells that affect tumor development, it was supposed that the opposite prognostic value of TILs in breast cancer may attribute to the complex interaction between cancer cells and immune cells. When the cancer cells are under the control of immune system, high level of TILs indicate better prognosis. On the contrary, immune cells in luminal breast cancer and DCIS are insufficient to affect tumor cells’ biological characteristics. Therefore, dense TILs are related to tumor cell’s aggressive features and patients’ poor prognosis. Some studies proposed that immune microenvironment may contribute to the ethnical disparity in breast cancer [26]. Compared with Western patients, the favorable prognosis in Asian patients may attribute to the abundance of TILs [27]. Regarding to DCIS, the prognostic value of TILs was observed in both Asian (Aye Aye THIKE et.al’s study [28]) and White (Farbod Darvishian et.al [11] and Elizabeth Thompson et.al’s study [9]). However, considering that the limited studies included in our meta-analysis display patients’ ethnicity, subgroup analysis was not conducted. Whether TILs and the compositions of TILs in DCIS exhibit significantly difference between patients with diverse ethnicity, and whether they have a diverse impact on patients’ mortality still remain to be investigated in the future.

Aside from total TILs, we also performed a meta-analysis for TIL subsets. Considering limited research using IHC staining to investigate subsets of TILs in DCIS, our meta-analysis is restricted. In our study, only CD4+ TILs and FOXP3+ TILs were marginally associated with the recurrence risk of DCIS. In addition to the T-TIL subset study contained in our meta-analysis, some studies also investigated B lymphocytes (TIL-Bs) and tumor-associated macrophages (TAMs), and tried to use the TIL ratio to assess DCIS recurrence risk. In I Miligy’s study, CD20+/CD19+ TIL-Bs were demonstrated to be associated with a shorter recurrence-free interval [29]; the prognostic value of CD68+ and CD163+ TAMs were also proposed in Xiao-Yang Chen [30] and Aye Aye Thike’s studies [31]. In addition, the ratio of FOXP3+/CD8+ and FOXP3+/CD4+ cells in Milim Kim’s study [18], and CD8HLADR+/− and CD115+ cells in Michael J. Campbell’s study [32], were associated with recurrence-free survival in DCIS. Although some of the studies observed positive results, related studies are still limited, and there is a lack of uniform and suitable assessment methods for the TIL ratio. Thus, no studies about TIL-B, macrophage and TIL ratios can be included in our meta-analysis. In recent years, accumulating evidence showed that PD-L1 expression is a novel prognostic marker for breast cancer [33]. In our subgroup analysis, it was also found that PD-L1 staining in DCIS is valuable, and stromal PD-L1 in immune cells is associated with recurrence risk closely than tumor cell PD-L1 staining. The limited cancer cells in DCIS exhibit PD-L1 expression, and PD-L1 staining in immune cells is partly positive, thus leading to the superior prognostic value of PD-L1 in TILs [7, 9].

Recent years, to optimize DCIS management, molecular assays about DCIS were created. Similar to the molecular assays of Oncotype DCIS score [3] and DCISionRT [20], TILs in DCIS could also provide valuable prognostic information about the risk of recurrence with low cost and convenience. Additionally, in our observation, TILs were not only suitable to patients who received solely surgery, but also to patients who were treated with surgery and radiotherapy. Therefore, TILs in DCIS may also be conductive to the guidance of the additional adjuvant therapies and follow-up. Though TILs have been described as reliable, reproducible and inexpensive markers in breast cancer, and could up−/ down-stage traditional pathological-staging in early-stage TNBC [21], limited research was focused on the utility of TILs in DCIS. On account of the convenience of TILs’ assessment, patients in clinical trials like ECOG-E5194 [22, 23] can be used to re-analyze the association between TILs and ipsilateral breast events. Meanwhile, investigating whether TILs in DCIS could be used to stratify patients with different risk along with traditional risk factors such as tumor grade, tumor size, and age is also meaningful. Aside from pathological detection, according to Tiantian Bian et.al’s study, the preoperative MRI-based radiomics signatures are valuable in evaluating the TILs level in breast cancer [34]. Currently, NCT03495011 trial is trying to investigate the MRI-based radiomics signatures that correlate with pathologic markers in DCIS. Therefore, it is meaningful to take TILs into concern in NCT03495011 trial, aiming to assess whether MRI signatures are related to the TIL level in DCIS. In the future, if MRI could also be used to assess TIL level in DCIS preoperatively and identify low-risk DCIS, a proportion of patients with low-risk DCIS may avoid surgery.

We made an effort to conduct a comprehensive review for the prognostic value of TILs and subsets of TILs in DCIS, but there are several limitations in our study. The main limitation of our study is that the eligible studies were relatively small, and all of the included records were retrospective studies. Our study is a literature-based analysis. The cutoff value in category patients with “dense TILs” and “sparse TILs” are not consistent between studies. We checked the publication bias with both Egger’s and Begg’s tests and did not observe an indication of publication bias. While studies with positive results were more prone to be published, relative data that did not observe a positive correlation between TILs and DCIS recurrence risk were partly unavailable [35, 36].

Taken together, our meta-analysis observed a potential prognostic role of tumor infiltrating lymphocytes (TILs) in DCIS and proposed that the assessment method of TILs and patient adjuvant therapies are critical in evaluating TIL prognostic value. In addition, we identified PD-L1 in TIL subsets as a potential marker in predicting the higher recurrence risk of DCIS. To achieve clinical translation, further prospective clinical trials investigating TILs in DCIS are needed in the future.

## Conclusions

For patients who received surgery or surgery accompanied with radiotherapy, dense stromal TILs in DCIS indicated higher recurrence risk. Among TIL subsets, dense stromal PD-L1+ TILs were valuable in predicting higher recurrence risk of DCIS.

## Data Availability

Not applicable.

## Abbreviations

TILs: Tumor infiltrating lymphocytes
t-TILs: Stromal touching TILs
p-TILs: Percentage of stromal TILs
TAMs: Tumor-associated macrophages
DCIS: Ductal carcinoma in situ
p-DCIS: Pure DCIS
i-DCIS: Pure DCIS mixed with IDC
m-DCIS: Pure DCIS mixed with microinvasive cancer
HE: Hematoxylin and eosin staining
IHC: Immunohistochemistry staining
TC: Tumor cell
S: Surgery
BCS: Breast conserving surgery
MAS: Mastectomy
AT: Adjuvant therapy
RT: Radiation therapy
HT: Hormonal therapy
IR: Ipsilateral recurrence
AR: All kinds of recurrence
SBE: Second breast cancer events
HR: Hazard ratio
CI: Confidence intervals
PRISMA: Preferred reporting item for systematic reviews and meta-analyses
Embase: Excerpta medica database
NOS: The newcastle-ottawa scale
UK: United Kingdom
USA: United States of America
